# Maturation and electrophysiological properties of human pluripotent stem cell‐derived oligodendrocytes

**DOI:** 10.1002/stem.2273

**Published:** 2016-01-13

**Authors:** Matthew R. Livesey, Dario Magnani, Elaine M. Cleary, Navneet A. Vasistha, Owain T. James, Bhuvaneish T. Selvaraj, Karen Burr, David Story, Christopher E. Shaw, Peter C. Kind, Giles E. Hardingham, David J.A. Wyllie, Siddharthan Chandran

**Affiliations:** ^1^ Centre for Integrative Physiology, University of EdinburghEdinburghUnited Kingdom; ^2^ Euan MacDonald Centre for MND Research, University of EdinburghEdinburghUnited Kingdom; ^3^ Centre for Clinical Brain Sciences, University of EdinburghEdinburghUnited Kingdom; ^4^ MRC Centre for Regenerative Medicine, University of EdinburghEdinburghUnited Kingdom; ^5^ Centre for Brain Development and Repair, Institute for Stem Cell Biology and Regenerative MedicineBangaloreKarnatakaIndia; ^6^ Maurice Wohl Clinical Neuroscience Institute, Institute of Psychiatry, Psychology and Neuroscience, King's College LondonDe Crespigny ParkLondonUnited Kingdom

**Keywords:** Stem cell, Oligodendrocyte, Electrophysiology, Human, Amyotrophic lateral sclerosis, oligodendrocyte precursor cell

## Abstract

Rodent‐based studies have shown that the membrane properties of oligodendrocytes play prominent roles in their physiology and shift markedly during their maturation from the oligodendrocyte precursor cell (OPC) stage. However, the conservation of these properties and maturation processes in human oligodendrocytes remains unknown, despite their dysfunction being implicated in human neurodegenerative diseases such as multiple sclerosis (MS) and amyotrophic lateral sclerosis (ALS). Here, we have defined the membrane properties of human oligodendrocytes derived from pluripotent stem cells as they mature from the OPC stage, and have identified strong conservation of maturation‐specific physiological characteristics reported in rodent systems. We find that as human oligodendrocytes develop and express maturation markers, they exhibit a progressive decrease in voltage‐gated sodium and potassium channels and a loss of tetrodotoxin‐sensitive spiking activity. Concomitant with this is an increase in inwardly rectifying potassium channel activity, as well as a characteristic switch in AMPA receptor composition. All these steps mirror the developmental trajectory observed in rodent systems. Oligodendrocytes derived from mutant *C9ORF72*‐carryng ALS patient induced pluripotent stem cells did not exhibit impairment to maturation and maintain viability with respect to control lines despite the presence of RNA foci, suggesting that maturation defects may not be a primary feature of this mutation. Thus, we have established that the development of human oligodendroglia membrane properties closely resemble those found in rodent cells and have generated a platform to enable the impact of human neurodegenerative disease‐causing mutations on oligodendrocyte maturation to be studied. Stem Cells *2016;34:1040–1053*

**Significance Statement**

Two main classes of cells are present in the brain ‐ neurons and glia. Both classes can be subdivided further and in the case of glia these fall into two main categories ‐ astrocytes and oligodendrocytes. Studies from rodents have indicated that as oligodendrocytes mature from their precursor cells they undergo changes such that they express different types and abundances of ion channels which are multimeric protein complexes that allow ions to pass across the cell membrane. Our study for the first time characterizes this process in human oligodendrocytes derived from pluripotent stem cells and serves not only to document this process in human cells but illustrates how this can be used to study diseases associated with oligodendrocyte dysfunction.

## Introduction

Dependent on context, it is increasingly recognized that oligodendrocytes, aside from their normal physiological function, can be injurious or regenerative in disease states. The regenerative potential of oligodendrocyte precursor cells (OPCs) is a major focus for research into demyelinating disease. Moreover, functional perturbations in oligodendrocyte differentiation and maturation from OPCs are implicated in disorders such as multiple sclerosis and amyotrophic lateral sclerosis (ALS) 1, 2. Oligodendrocyte pathology ranging from inclusions and myelin abnormalities to reactive changes in OPCs is described in ALS 3, 4, 5. Demyelination is also present in the most common genetic cause of ALS due to a hexanucleotide intronic repeat in the *C9ORF72* gene that accounts for approximately 10%–50% of familial and around 6%–10% of sporadic cases across European populations 3, 6, 7, 8. However, the differentiation and maturation potential of *C9ORF72* repeat expansion‐carrying OPCs has not been investigated before. Improved mechanistic understanding of the differentiation and maturation of human oligodendrocytes from OPCs is therefore of considerable interest as a point of potential therapeutic intervention. Specifically the excitable membrane properties of oligodendrocytes that are central to their physiological role have been shown in rodent studies to markedly change upon differentiation and maturation of the OPC to an oligodendrocyte. The necessity to explore oligodendrocyte maturation in a human context is underlined by evidence for both interspecies cellular and molecular differences in neurons and astrocytes 9, 10, 11, 12, 13 as well as specific differences in the spatial distribution and development of rodent and human oligodendrocytes 14. However, the conservation of excitable membrane properties throughout the maturation process in human oligodendrocytes remains unknown, despite their dysfunction being implicated in diseases such as ALS. The ability to derive oligodendrocyte lineage cells from human pluripotent stem cells (hPSCs) including from patients carrying disease causing mutations provides an opportunity to investigate the maturation and electrophysiological properties of human oligodendrocytes in both normal physiological and disease contexts 15, 16, 17.

Here, we have designed a protocol that reliably generates a scalable population of OPCs from hPSCs that upon differentiation yields cultures enriched for oligodendrocytes, enabling us to study the maturation and physiological properties of oligodendrocytes. Using a combination of electrophysiological, biochemical, and immunohistochemical approaches, we show species conservation of the defining physiological properties of differentiated oligodendrocytes that are distinct from those of OPCs. OPCs derived from mutant *C9ORF72*‐carrying ALS patient induced pluripotent stem cells (iPSCs) did not exhibit impairment to maturation or differences in viability despite the presence of key pathological features, including RNA foci, suggesting that maturation defects may not be a primary feature of this mutation.

## Material and Methods

### OPC and Oligodendrocyte Production

The SHEF4 line (male, UK Stem Cell Bank), referred to as the “ES line”, was obtained under full Ethical/Institutional Review Board approval of the University of Edinburgh. Written informed consent was obtained from each participant providing the lines iPS1 (female), iPS2 (male), iPS^C9^1 (female), and iPS^C9^2 (male). The derivation, characterization, and validation of iPS lines were performed as described previously 18 and are detailed in the Supporting Information Text. For neuralization, spontaneous embryo body formation was induced following cell lifting with dispase (17105‐041, Life Technologies, CA, USA) and collagenase (17104‐019, Life Technologies). Embryo bodies were cultured for 7 days, with dual‐SMAD inhibition 19, and in chemically defined media (CDM) that contained 50% Iscove's modified Dulbecco's medium (Invitrogen, CA, USA, 50% F12, BSA (5 mg/ml, Europa, Cambridge, UK), 1% chemically defined Lipid 100 (Invitrogen), monothioglycerol (450 μM, Sigma‐Aldrich, Gillingham, UK), insulin (7mg/ml), Roche, Basel, Switzerland), transferrin (15 mg/ml, Roche), 1% penicillin/streptomycin), supplemented with N‐acetyl cysteine (1 mM, Sigma), activin Inhibitor (10 μM, R&D Systems, MN, USA), and Dorsomorphin (2 μM, Merck Millipore, Darmstadt, Germany). NPCs were cultured in suspension as neurospheres with media changes every 2–3 days. Neurospheres were then caudalized by the addition of retinoic acid (1 μM, Sigma) for a further 7 days. At this stage, neuropheres were quality controlled for their morphology and the formation neural rosettes before subsequently being ventralized with the addition of the sonic hedgehog agonist purmorphamine (1 μM, Calbiochem, CA, USA) for 7 days in Advanced DMEM/F12 (Invitrogen) containing: 1% N‐2 supplement (Invitrogen), 1% B27 supplement (Invitrogen), 1% penicillin/streptomycin (Invitrogen), 0.5% GlutaMAX (Invitrogen), and 5 μg/ml heparin (Sigma). Ventral spinal cord patterned progenitor cells were then further expanded in the presence of basic fibroblast growth factor (FGF)‐2 (10 ng/ml, PeproTech, NJ, USA) for 7 days. Subsequently, neuronal and glia differentiation of NPCs was triggered by FGF2 withdrawal for a further 2 weeks. At this stage neurospheres‐containing OLIG2^+^ cells were further expanded for additional 2 weeks in the presence of FGF2 (10 ng/ml), PDGFα (Platelet‐derived growth factor alpha, 20 ng/ml, PeproTech), purmorphamine (1 μM), and SAG (1 μM, Calbiochem), IGF‐1 (10 ng/ml, PeproTech), T3 (60 ng/ml, Sigma), and 1 × ITS (Gibco, NY, USA) before initiating oligodendrocyte differentiation. It was possible to maintain OPC‐containing spheres in this media for up to 8 weeks. Terminal differentiation of oligodendrocytes was achieved by mitogen withdrawal (except for T3 and IGF‐1) following single‐cell dissociation using papain (Worthington Biochemical, NJ, USA) and plating on Matrigel (SLS, 354230; 1 in 100 dilution), fibronectin (20 μg/ml, F2006, Sigma‐Aldrich), laminin (10 μg/ml, L2020, Sigma‐Aldrich) coated coverslips (or plates) at a density of 20,000‐30,000 cells per 0.3 cm^2^. For biochemical studies, cultures were dissociated with accutase (Life technologies), and separation of O4^+^‐oligodendrocytes was achieved by magnetic‐activated cell sorting (MACS) using anti‐O4 MicroBeads (MACS 130‐096‐670) and MACS LS columns (MACS 130‐042‐401) following the manufacturer's instructions.

### Immunocytochemistry

All steps were performed at 18–24°C. Cells were fixed with 4% paraformaldehyde in phosphate‐buffered saline (PBS) for 20 minutes, permeabilized with 0.2% Triton X‐100 containing PBS, then blocked in 3% goat serum, then incubated with appropriate primary and secondary antibodies. Nuclei were counterstained with either DAPI (4′,6‐diamidino‐2‐phenylindole, Sigma‐Aldrich) or Hoechst staining (bisbenzimide; Sigma), and coverslips were mounted on slides with FluorSave (Merck, NJ, USA). Fluorescent imaging was performed using an Axioscope (Zeiss, Oberkochen, Germany) microscope. Images were processed with Axiovision V 4.8.1 (Zeiss). Regions of interest were selected based on uniform DAPI staining and imaged for two to four fluorescent channels, and immunolabeled cells counted manually with ImageJ64 (v 1.47) software for an area of 162.40 μm^2^. Positive staining was defined as a signal clearly above the background fluorescence present in areas devoid of cells. At least three independent images per culture and at least three independent cultures from three independent conversions were analyzed.

Live‐staining was performed with the addition of 150 μl of Ο4 antibody (1:300, R&D Systems) or platelet‐derived growth factor alpha (PDGFRα) (1:200 Cell Signaling, MA, USA) in culture media to 100 μl of culture media left in each 24‐well plate. Cells were incubated at 37°C for 40 minutes after which coverslips were washed with media and stained with secondary antibody diluted in media (1:1,000). Cells were incubated at 37°C for a further 30 minutes, washed, and then assessed electrophysiologically.

### Electrophysiology

The whole‐cell patch configuration was used to record macroscopic currents as described previously 20, 21. Reported potential values are corrected for liquid junction potential (+14 mV). Responses to α‐amino‐3‐hydroxy‐5‐methyl‐4‐isoxazolepropionic acid (AMPA) and *N*‐methyl‐D‐aspartate (NMDA) were recorded at –74 mV and responses to gamma‐aminobutyric acid (GABA) and glycine at –14 mV. Current and voltage measurements were typically low‐pass filtered online at 2 kHz, digitized at 10 kHz, and recorded to computer using the WinEDR V2 7.6 Electrophysiology Data Recorder (J. Dempster, Department of Physiology and Pharmacology, University of Strathclyde, UK; www.strath.ac.uk/Departments/PhysPharm/).

### Statistical Analysis

Data are presented as mean ± SEM. Rectification indices (RI) were calculated from the following equation:

$$\text{RI}=\frac{\left[I/\left(16-E_{\text{REV}}\right)\right]}{\left[I/\left(-124-E_{\text{REV}}\right)\right]}$$

where *I* represents current amplitude and *E*_*REV*_ indicates the reversal potential of currents. The number of experimental replicates is denoted as “*n*” while “*N*” represents number of de novo preparations from which *n* is obtained. The Shapiro‐Wilk test was used to assess whether data were normally distributed and then either a Student's *t* test or a Mann Whitney *U* test were used to determine statistical significance with *, *p < * 0.05; **, *p < * 0.01; and ***, *p < * 0.001.

Details of EdU labeling and detection, flow cytometry, quantitative polymerase chain reaction (qPCR), and RNA fluorescence in situ hybridization (FISH) methodologies are included in the Supporting Information Text.

## Results

### Derivation of OPCs and Oligodendrocytes from Control hPSCs

**Figure 1:**
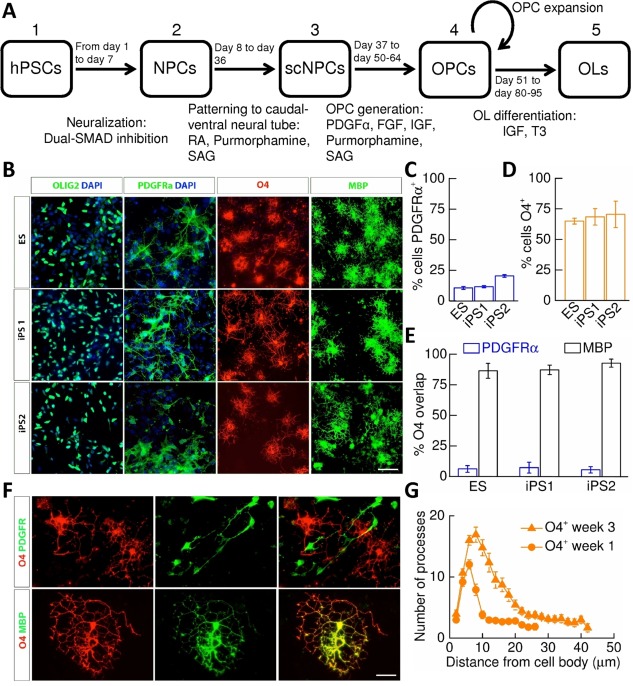
Derivation and specification of oligodendrocytes from human pluripotent stem cell (hPSC)‐derived oligodendrocyte precursor cells (OPCs). **(A)**: Summary of the protocol used to generate hPSC‐derived oligodendrocytes: **(1)** hPSCs were neuralized via dual‐SMAD inhibition. **(2)** NPCs were patterned to ventral spinal cord by exposure to retinoic acid and sonic hedgehog agonists, purmorphamine, and SAG. **(3)** spinal cord‐patterned NPCs were converted to OPCs by exposure to PDGFα and other mitogens. **(4)** OPCs could be further expanded by mechanical dissociation. **(5)** Oligodendrocyte differentiation was induced by mitogen withdrawal. **(B)**: Representative staining of cells 1 week post‐mitogen removal identifies OLIG2^+^‐progenitors, and cells expressing PDGFRα, O4, and MBP in all lines examined. Scale bar = 50 μm. **(C)**: Percentage of PDGFRα^+^‐cells (ES/iPS1/iPS2: *N* = 4/4/3) and **(D)** O4^+^‐cells (ES/iPS1/iPS2: *N* = 4/13/13) in week 1 cultures. **(E)**: Percentage O4^+^‐cells that express PDGFRα(ES/iPS1/iPS2: *N* = 4/4/3) and MBP (ES/iPS1/iPS2: *N* = 4/5/6). Equivalent cellular specification was observed across all lines. **(F)**: Example images illustrating no overlap of PDGFRα^+^‐ and O4^+^‐cells (upper panels) but substantial overlap of MBP^+^‐ and O4^+^‐cells (lower panels). Scale bar = 12 μm. **(G)**: Sholl analysis performed upon week 1 and 3 O4^+^‐oligodendrocytes. Abbreviations: DAPI, 4',6‐diamidino‐2‐phenylindole; ES, embryonic stem cell; FGF, fibroblast growth factor; hPSC, human pluripotent stem cell; IGF, Insulin‐like growth factor; iPS, induced pluripotent stem cell; MBP, myelin basic protein; NPC, neural precursor cell; OL, oligodendrocyte; OPC, oligodendrocyte precursor cell; PDGF, platelet‐derived growth factor; RA, retinoic acid; SAG, smoothened agonist; scNPC, spinal cord‐patterned neural precursor.

We first optimized a protocol to generate enriched in vitro cultures of control oligodendrocytes from three hPSC lines; one embryonic stem cell (ESC) line and two iPSCs (iPS1, iPS2; summarized in Fig. 1A). In brief, expansion of OLIG2^+^ retinoic acid‐ and purmorphamine‐treated NPCs 18 in the presence of FGF and PDGF resulted in conversion into OPCs that were positive for PDGFRα 22 over 2–4 weeks. Plate‐down and withdrawal of mitogens resulted in oligodendrocyte differentiation. Quantitative immunocytochemistry performed 1 week after differentiation (Fig. 1B–1D) revealed that cultures gave rise to a majority of O4^+^‐labeled oligodendendrocytes (ESC, 65.0 ± 2.4%; iPS1, 68.5 ± 6.8%; iPS2, 70.5 ± 10.8%) with a residual population of PDGFRα^+^‐OPCs (ESC, 10.5 ± 1.1%; iPS1, 11.6 ± 0.9%; iPS2, 20.3 ± 1.1%). Notably, O4^+^‐oligodendrocytes exhibited high coexpression of myelin basic protein (MBP) (ESC, 86.5 ± 6.1%; iPS1, 87.2 ± 3.9%; iPS2, 92.7 ± 3.3%) and very little overlapping PDGFRα expression (ESC, 6.1 ± 2.7%; iPS1, 7.2 ± 4.5%; iPS2, 5.4 ± 2.6%; Fig. 1E, 1F). By week 3 of differentiation, the number of O4^+^ cells remained at levels similar to those seen at week 1 across all lines (*p* ≥ 0.3, unpaired *t* tests). PDGFRα^+^‐OPCs and O4^+^/MBP^+^‐oligodendrocytes displayed distinct morphology, with OPCs being morphologically less complex whereas oligodendrocytes possessed a radial and multibranched morphology (Fig. 1F). Sholl analysis also revealed an increase in number and complexity of O4^+^/MBP^+^ oligodendrocyte processes from week 1 to 3 (Fig. 1G).

### Maturation of hPSC‐Derived Oligodendrocyte‐Lineage Membrane Currents

**Figure 2:**
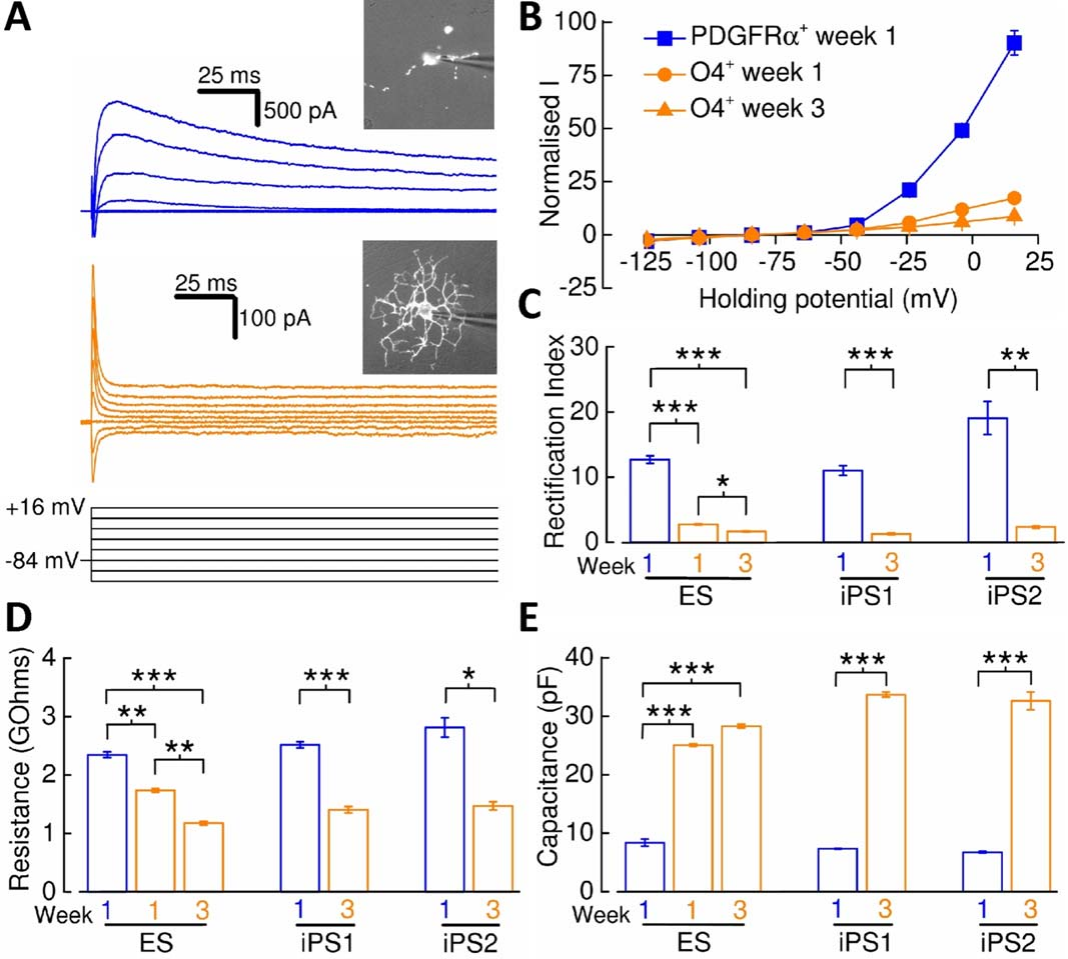
Membrane current properties of human pluripotent stem cell‐derived oligodendrocyte precursor cells (OPCs) and oligodendrocytes. **(A)**: Whole‐cell current recordings from a PDGFRα^+^‐OPC (blue) and week 3 O4^+^‐oligodendrocyte (orange) in response to a voltage‐step protocol that involved incremental application of 20 mV voltage steps from a holding potential of –84 mV. Live‐stained cells are shown inset. **(B)**: Mean normalized current–voltage plots for PDGFRα^+^‐OPCs, week 1 (circles) and week 3 (triangles) O4^+^‐oligodendrocytes (*n* = 9–17, *N* = 3–6) derived from the ES line. Current amplitudes were measured 175 milliseconds after voltage‐step initiation and normalized to –64 mV current data. **(C)**: Mean PDGFRα^+^‐OPC (iPS1/iPS2: *n* = 10/5, *N* = 3/1) and week 3 O4^+^‐oligodendrocyte (iPS1/iPS2: *n* = 8/6, *N* = 4/2) rectification index data calculated for each line examined. **(D)**: Mean input resistance measurements for PDGFRα^+^‐OPCs (ES/iPS1/iPS2: *n* = 20/21/8, *N* = 3/3/3) and O4^+^‐oligodendrocytes (ES/iPS1/iPS2: *n* = 26‐32/19/7, *N* = 3/3/3). **(E)**: Mean whole‐cell capacitance measurements for PDGFRα^+^‐OPCs (ES/iPS1/iPS2: *n* = 22/21/12, *N* = 3/3/3) and O4^+^‐oligodendrocytes (ES/iPS1/iPS2: *n* = 32‐41/26/7, *N* = 3/3/3). *, *p* < 0.05; **, *p* < 0.01, ***, *p* < 0.001, respectively. Abbreviations: ES, embryonic stem cell; iPS, induced pluripotent stem cell; PDGFRα, platelet‐derived growth factor receptor alpha.

To address whether human OPCs and oligodendrocytes exhibit conservation of cell‐type specific membrane conductances in response to membrane depolarization 23, 24, 25, we undertook electrophysiological recordings from OPCs and cells colabeled for O4 and MBP. Having already established extremely limited colabeling of PDGFRα and O4 (Fig. 1C, 1E), electrophysiological recordings were undertaken on live‐stained OPCs and differentiated oligodendrocytes using antibodies against PDGFRα and O4 (Fig. 2A). For PDGFRα^+^‐OPCs at week 1, a depolarizing voltage‐step protocol induced large outward currents that consisted of transient and sustained components (Fig. 2A). In contrast, the application of the same voltage‐step protocol applied to O4^+^‐oligodendrocytes at week 1 and 3 yielded currents that were substantially lower in amplitude than those observed in OPCs (Fig. 2A). Mean current–voltage (I–V) relationships constructed from this data show that evoked membrane currents in hPSC‐derived OPCs are outwardly rectifying and differentiation to O4^+^‐oligodendrocytes is associated with a reduced degree of outward rectification (Fig. 2B). Rectification indices were subsequently calculated (see Materials and Methods section) to quantify such rectification shifts and revealed that current rectification was reduced in each line of week 3 O4^+^‐oligodendrocytes when compared with the levels seen in PDGFRα^+^‐OPCs (ESC, 13.4 ± 2.2%; iPS1, 11.8 ± 3.8%; iPS2, 12.6 ± 2.5%, Fig. 2C). A reduction in input resistance also accompanies this shift in membrane current properties (Fig. 2D) therefore providing added support to the notion that changes in ion channel expression accompany oligodendrocyte differentiation from hPSC‐derived OPCs. Furthermore, whole‐cell capacitance measurements are consistent that the membrane compartment increases in size during the oligogodendrocyte differentiation process (Fig. 2E). Together these data indicate that maturation is active in differentiated oligodendrocytes.

### Distinctive Maturation‐Specific Changes in Functional Ion Channel Expression in hPSC‐Derived Oligodendrocyte‐Lineage Cells

**Figure 3:**
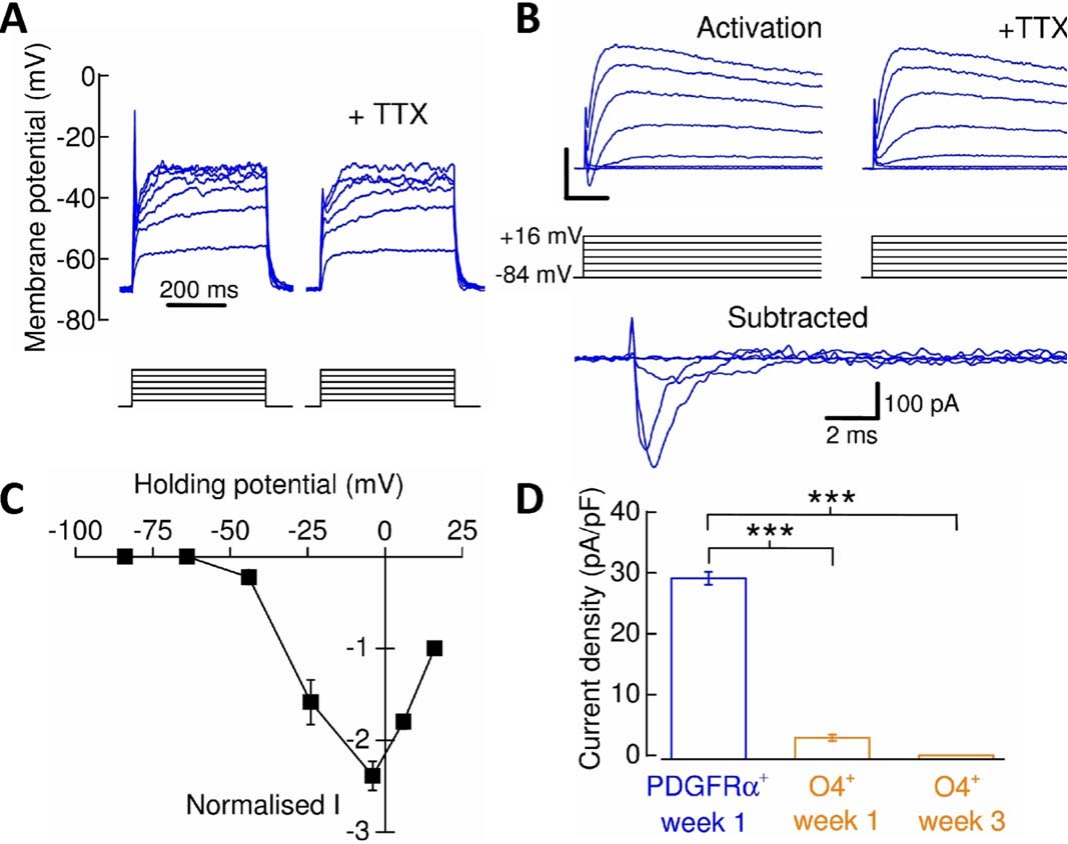
Voltage‐gated Na^+^‐channel expression in human pluripotent stem cell‐derived oligodendrocyte precursor cells (OPCs) and oligodendrocytes. **(A)**: Current‐clamp recording demonstrating that PDGFRα^+^‐OPCs exhibited tetrodotoxin (TTX)‐sensitive spikes in response to depolarization by current injection (each current step below trace represents 10 pA). **(B)**: To isolate and measure *Na*_*V*_‐channel activity, the membrane potential was initially stepped in 20 mV increments from –84 mV to + 16 mV (*activation*). Scale bars = 500 pA, 5 milliseconds. The protocol was then repeated in the presence of TTX and the current data subtracted from that of the former to yield the TTX‐sensitive *Na*_*V*_‐specific current. Not all TTX‐sensitive currents are shown for figure clarity. **(C)**: Normalized current–voltage plot of *Na*_*V*_‐channel activity expressed by PDGFRα^+^‐OPCs. Data were normalized to +16 mV current data. **(D)**: Decrease in *Na*_*V*_‐channel expression from PDGFRα^+^‐OPCs to O4^+^‐oligodendrocytes (*n* = 5–18, *N* = 3; Mann Whitney *U* tests). ***, *p* < 0.001. Abbreviations: PDGFRα, platelet‐derived growth factor receptor alpha; TTX, tetrodotoxin.

Rodent OPCs and oligodendrocytes exhibit cell‐type specific voltage‐gated ion channel expression profiles, which are correlated to developmentally defined oligodendroglial immunohistochemical markers and underpin the properties of the membrane currents described above 24, 25. Depolarizing current injections applied to PDGFRα^+^‐OPCs gave rise to tetrodotoxin (TTX, 300 nM) sensitive spikes (16 from 18 cells) and indicate the functional expression of voltage‐gated Na^+^ (*Na*_*V*_) channels (Fig. 3A). As has been reported for rodent OPCs 26 spiking behavior did not fit the criteria that would be used to classify neuronal action potential firing as these spike amplitudes did not generally cross 0 mV. The existence of native spiking and non‐spiking OPC populations has been subject of numerous recent studies 26, 27, 28, and we therefore further isolated and characterized the *Na*_*V*_‐channels expressed in hPSC‐oligodendroglial cells using a depolarizing voltage‐step protocol (Fig. 3B, 3C). We found 89% of ESC‐ and 100% of iPSC‐derived PDGFRα^+^‐OPCs possessed TTX‐sensitive currents. However, all functional TTX‐sensitive *Na*_*V*_‐channel expression (current density) is lost in week 3 O4^+^‐oligodendrocytes derived from the ES line (Fig. 3D) and iPS lines (data not shown).

**Figure 4:**
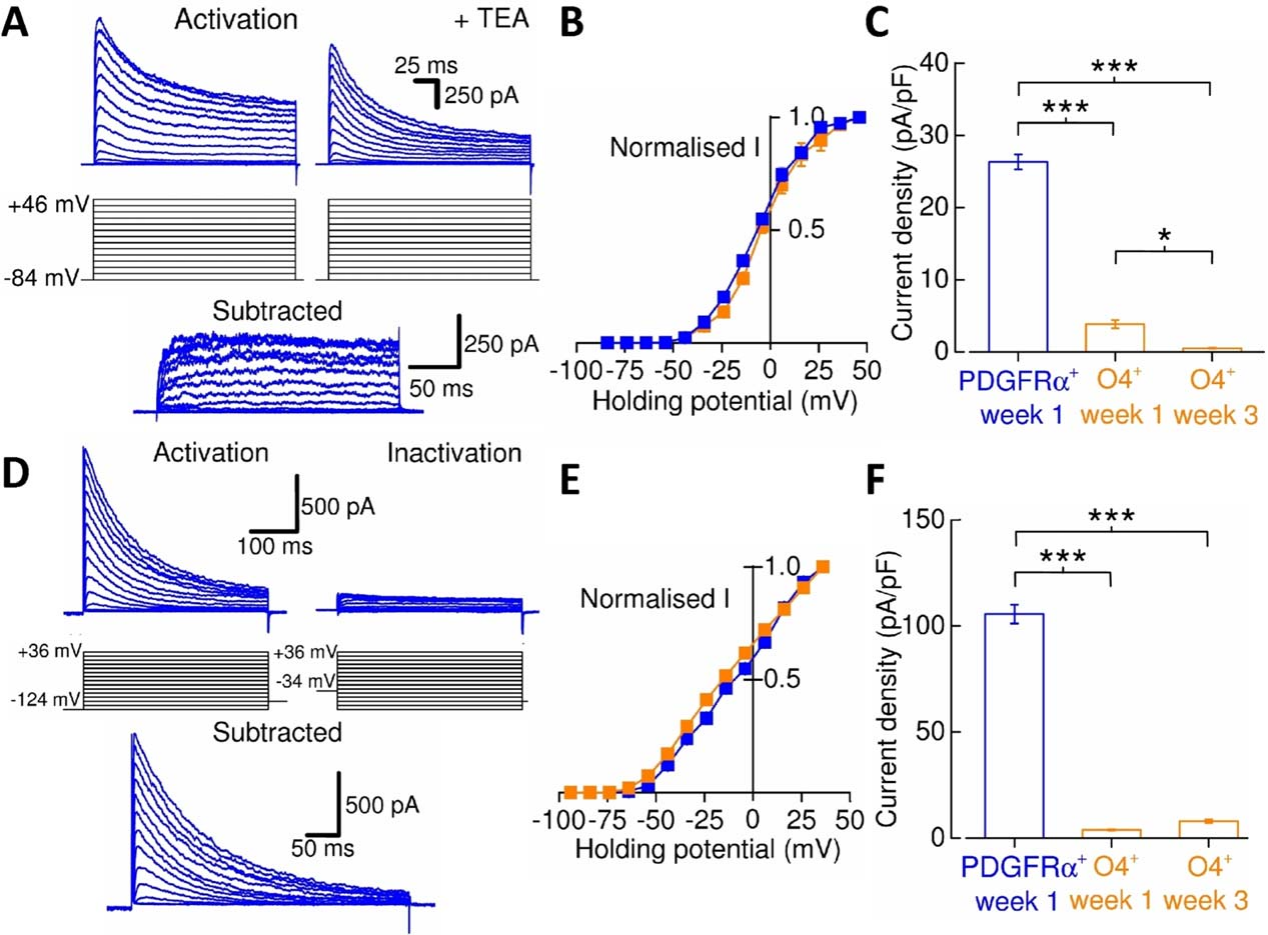
Voltage‐gated K^+^‐channel expression in human pluripotent stem cell‐derived oligodendroglia. **(A)**: To isolate *I*_*k*_‐channel activity, 10 mV incremental voltage‐pulses were initially applied to activate *I*_*k*_‐channels in the presence of tetrodotoxin (TTX) (*activation*). This was then repeated in the presence of TEA and the current data subtracted from that of the former to determine the *I*_*K*_‐specific current (*subtracted*). *I*_*K*_‐current amplitudes were measured 200 milliseconds after activation. The examples shown are from PDGFRα^+^‐oligodendrocyte precursor cells (OPCs). **(B)**: Normalized current–voltage plot of *I*_*K*_‐channel activity measured from PDGFRα^+^‐OPCs (*n* =  4) and O4^+^‐oligodendrocytes (*n* =  4). Data were normalized to +46 mV current data. **(C)**: Decrease in *I*_*K*_‐channel expression from PDGFRα^+^‐OPCs to O4^+^‐oligodendrocytes (*n* = 8–15, *N* = 3‐4). Current amplitude data were measured from the 100‐mV step. **(D)**: *I*_*A*_‐channel activity was measured in the presence of TTX and Cd^2+^ (100 μM). The holding potential was pre‐stepped to –124 mV (500 milliseconds) and, there from, the holding potential depolarized in 10 mV increments before returning to –84 mV (*activation*). Since *I*_*A*_‐channels inactivate rapidly upon depolarization, an inactivation protocol pre‐stepped cells to –34 mV from –84 mV to isolate non‐*I*_*A*_ current (*inactivation*), which was subtracted from the former to generate the *I*_*A*_‐mediated current (*subtracted*). *I*_*A*_‐current amplitudes were measured from the transient peak responses. The examples shown are from PDGFRα^+^‐OPCs. **(E)**: Normalized current–voltage plot of *I*_*A*_‐channel activity measured from PDGFRα^+^‐OPCs (*n* = 6) and O4^+^‐oligodendrocytes (*n* = 4). Data were normalized to +36 mV current data. **(F)**: Decrease in *I*_*A*_‐channel expression from PDGFRα^+^‐OPCs to O4^+^‐oligodendrocytes (*n* = 6‐12, *N* = 3‐4). Current amplitude data were measured from the depolarization step to +16 mV. *, *p* < 0.05; ***, *p* < 0.001. Abbreviations: PDGFRα, platelet‐derived growth factor receptor alpha; TEA, tetraethyl ammonium.

In rodent OPCs delayed outwardly rectifying K^+^ (*I*_*K*_) channels and A‐type K^+^ (*I*_*A*_) channels largely contribute to sustained and transient outward membrane currents, respectively, and are downregulated upon differentiation to oligodendrocytes 24, 25. Analysis of the expression of *I*_*K*_‐mediated currents (Fig. 4A, 4B) in OPCs and oligodendrocytes derived from the ES line revealed that there was a greater than 50‐fold reduction in *I*_*K*_‐channel current density in week 3 O4^+^‐oligodendrocytes compared with the levels seen in week 1 PDGFRα^+^‐OPCs (Fig. 4C). *I*_*A*_‐channel activity (Fig. 4D, 4E) was also reduced as cells differentiated from PDGFRα^+^‐OPCs to O4^+^‐oligodendrocytes (Fig. 4F). This strong downregulation in week 3 O4^+^‐oligodendrocytes of *I*_*K*_ (1.9 ±  0.8% of the value seen in week 1 PDGFRα^+^‐OPCs) and *I*_*A*_ (7.6 ± 1.9%) seen in the ES line was recapitulated in the two iPS lines (For *I*_*K*_; iPS1, 2.9 ± 1.2%, iPS2, 7.0 ± 1.1%, *n* = 4–6, *N* = 2, and for *I*_*A*_; iPS1, 0.8 ± 0.4%, iPS2, 5.0 ± 0.5%, *n* = 3–9, *N* = 2–9, *p*<0.001 in all cases, Mann‐Whitney *U* tests). *I*_*A*_ and *I*_*K*_ currents were detected in all PDGFRα^+^‐OPCs. In ESC‐derived week 3 O4^+^‐oligodendrocytes expression of *I*_*A*_ and *I*_*K*_ currents were observed in 78% and 83% of cells, respectively, whereas in iPSC‐derived week 3 O4^+^‐oligodendrocytes *I*_*A*_ and *I*_*K*_ currents were seen in 83% and 67% of cells, respectively.

**Figure 5:**
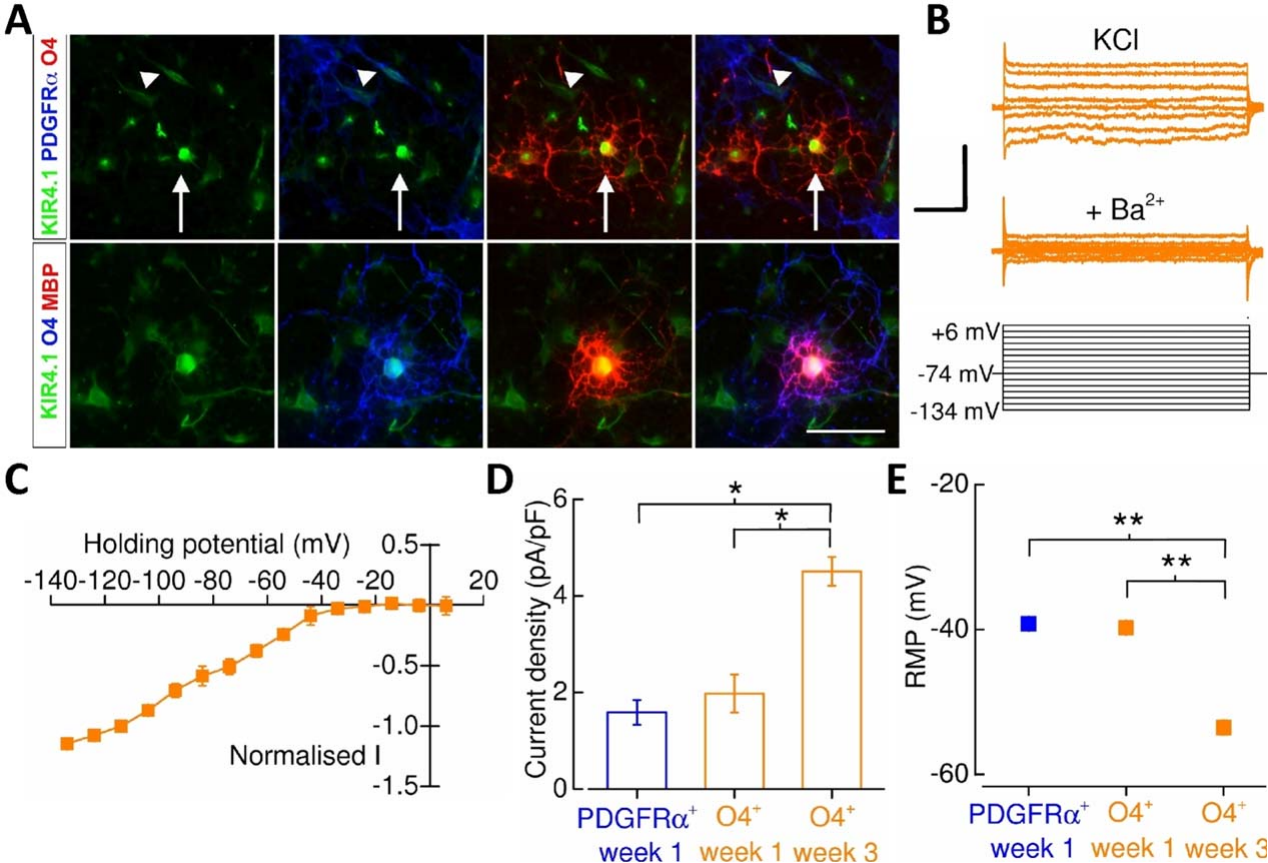
Inwardly rectifying K^+^‐channel expression in human pluripotent stem cell‐derived oligodendrocyte precursor cells (OPCs) and oligodendrocytes. **(A)**: Representative images of *K*_*ir*_4.1 subunit immunostaining in PDGFRα^+^‐OPCs (*arrowhead*), and O4^+^‐ (*arrow*) and MBP^+^‐oligodendrocytes. Scale bar = 30 μm. **(B)**: *K*_*ir*_‐channel measurements were performed using an extracellular solution in which KCl (50 mM) replaced an equimolar amount of NaCl. To isolate of *K*_*ir*_‐channel activity initially 10 mV incremental voltage‐steps were applied in the range –134 mV to + 6 mV from a holding potential of –74 mV and then repeated in the presence of Ba^2+^ (1 mM). Note that only selected voltage‐step current recordings are shown in the figure for clarity. Leak‐subtraction of *K*_*ir*_‐channel current data were performed using the pre‐pulse current amplitude in the presence of Ba^2+^ as zero current. It was not possible to extract a current–voltage (I‐V) plot from PDGFRα^+^‐cells given the very low *K*_*ir*_ channel current amplitudes. Scale bars = 100 pA, 50 milliseconds. **(C)**: Normalized I‐V of *K*_*ir*_‐channel activity obtained from O4^+^‐oligodendrocytes (*n* = 7). Data were normalized to –114 mV current data. **(D)**: An increase in mean *K*_*ir*_‐channel expression in week 3 O4^+^‐oligodendrocytes (*n* = 8, *N* = 3) from that of week 1 O4^+^‐oligodendrocytes (*n* = 10, *N* = 2) and PDGFRα^+^‐OPCs (*n* = 9, *N* = 3). Current amplitude data were measured from the depolarization step to –134 mV. Note that the increase in current density also factors the whole‐cell capacitance. **(E)**: The mean resting membrane potential PDGFRα^+^‐OPCs (*n* = 19, *N* = 3), week 1 O4^+^‐oligodendrocytes (*n* = 29, *N* = 7) and week 3 O4^+^‐oligodendrocytes (*n* = 20, *N* = 4). Error bars are obscured by the mean data point. *, *p* < 0.05; **, *p* < 0.01, respectively. Abbreviations: PDGFRα, platelet‐derived growth factor receptor alpha; RMP, resting membrane potential.

In contrast to decreases in *I*_*K*_‐ and *I*_*A*_‐channel expression, differentiation of rodent oligodendrocytes from OPCs is associated with an increase in inwardly rectifying K^+^ (*K*_*ir*_) channel expression 24. Specifically the *K*_*ir*_4.1 subunit has been reported to play a pivotal role in oligodendrocyte development, myelination and setting the resting membrane potential (RMP) of native mature oligodendrocytes 29 but see 30. There are no selective blockers that can be used to isolate pharmacologically *K*_*ir*_4.1‐mediated currents and therefore we confirmed immunohistochemically the presence of *K*_*ir*_4.1 subunits in O4^+^/MBP^+^‐oligodendrocytes and PDGFRα^+^‐OPCs (Fig. 5A). Subsequent analysis of *K*_*ir*_ channel expression in the ES‐line (Fig. 5B, 5C) indicated an increase in inwardly rectifying current densities in week 3 oligodendrocytes compared with both week 1 oligodendrocytes and PDGFRα^+^‐OPCs (Fig. 5D). This was also accompanied by an increase in the detection of *K*_*ir*_ currents (56% in PDGFRα^+^‐OPCs and 100% in week 3 O4^+^‐oligodendrocytes). Associated with this, we observed a hyperpolarization of the RMP from –39.2 ± 0.4 mV in OPCs to –53.5 ± 1.0 mV in week 3 oligodendrocytes (Fig. 5E). Thus, the maturation of human oligodendrocytes displays a comparable profile of potassium channel conductances to that seen in rodents.

### Expression of Neurotransmitter Ligand‐Gated Ion Channels in hPSC‐Derived Oligodendroglial Cells

**Figure 6:**
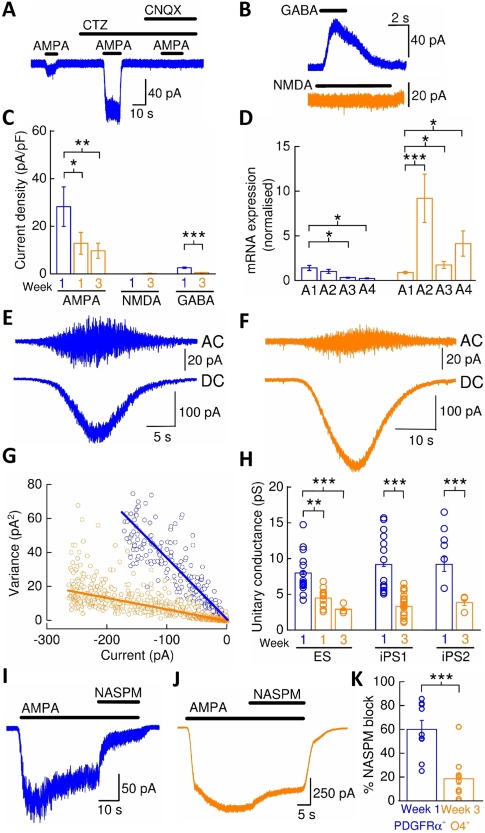
AMPA receptors in human pluripotent stem cell (hPSC)‐derived oligodendrocyte precursor cells (OPCs) and oligodendrocytes**. (A)**: AMPA‐mediated whole‐cell current responses in a PDGFRα^+^‐OPC are strongly potentiated by cyclothiazide and blocked by CNQX. **(B)**: Depicts sample recordings in which responses to applications of GABA (100 μM), NMDA (100 μM, in the presence of glycine [50 μM]) were obtained from week 3 O4^+^‐oligodendrocytes. **(C)**: Mean current densities for AMPA (*n* = 10/7/11, *N* = 2/1/2), NMDA (*n* = 6/8, *N* = 2/2), and GABA responses (*n* = 8/19, *N* = 3/3). **(D)**: Mean normalized mRNA fold expression data for AMPAR subunits GluA1‐GluA4 in OPC cultures (*N* = 3) and week 3 O4^+^‐oligodendrocytes (*N* = 13) as assessed by quantitative real‐time polymerase chain reaction. Data were normalized to GluA1 for each maturation stage after normalizing to β‐actin and GAPDH. **(E)**: Sample nonstationary fluctuation analysis recordings of AMPAR‐mediated currents from a PDGFRα^+^‐OPC and, **(F)** week 3 O4^+^‐oligodendrocyte. **(G)**: Plot describing the linear relationship of the variance of the AC‐coupled current to the DC‐current amplitude for the former recordings in *c* and *d*. The fitted slopes for each plot gave respective unitary single‐channel current amplitude estimates of −0.45 pA and −0.1 pA, respectively, from which the unitary conductance was calculated. **(H)**: Mean AMPAR conductance for PDGFRα^+^‐OPCs and O4^+^‐oligodendrocytes derived from control hPSC lines. **(I)**: Example recording of 1‐naphthyl acetyl spermine (NASPM) block of steady‐state currents evoked by AMPA in a PDGFRα^+^‐OPC and, **(J)** week 3 O4^+^‐oligodendrocyte. **(K)**: Mean percentage block of AMPA currents by NASPM. *, *p* < 0.05; **, *p* < 0.01; ***, *p* < 0.001, respectively. Abbreviations: AMPA, α‐amino‐3‐hydroxy‐5‐methyl‐4‐isoxazolepropionic acid; CNQX, 6‐cyano‐7‐nitroquinoxaline‐2,3‐dione; CTZ, cyclothiazide; GABA, gamma‐aminobutyric acid; NASPM, 1‐naphthyl acetyl spermine; NMDA, *N*‐methyl‐D‐aspartate; PDGFRα, platelet‐derived growth factor receptor alpha.

Ligand‐gated ion channels (LGICs) for a variety of neurotransmitters play major roles in oligodendroglial‐lineage cell physiology and pathophysiology 31 and we therefore examined the functional expression of AMPARs, GABA_A_Rs, NMDARs, and strychnine‐sensitive glycine receptors (GlyRs) in our cultures. Application of AMPA (10 μM) elicited small inward currents in PDGFRα^+^‐OPCs which could be potentiated (1,320 ± 500%; *n* = 5) in the presence of the AMPAR selective potentiator, cyclothiazide (10 μM; Fig. 6A). The AMPAR antagonist 6‐cyano‐7‐nitroquinoxaline‐2,3‐dione (CNQX) (30 μM) blocked these agonist‐evoked currents. All cells examined responded to AMPA, although AMPAR current densities decreased as OPCs matured to oligodendrocytes (Fig. 6C). Responses to NMDA (100 μM, in the presence of glycine (50 μM)) were not observed in either PDGFRα^+^‐OPCs (*n* = 6) or week 3 O4^+^‐oligodendrocytes (*n* = 8; Fig. 6B, 6C). GABA (100 μM) evoked responses in 63% in PDGFRα^+^‐OPCs (*n* = 8) and 74% in O4^+^‐oligodendrocytes (*n* = 19) which were blocked by picrotoxin. GABA_A_R current densities decreased during OPC maturation (Fig. 6B, 6C). Applications of glycine (100 μM) did not elicit responses in PDGFRα^+^‐OPCs or week 3 O4^+^‐oligodendrocytes (data not shown).

### Expression of Arginine‐Edited GluA2 AMPAR Subunits Is Upregulated in hPSC‐Derived Oligodendrocytes

The expression and activation of OPC AMPARs is considered crucial to OPC physiology and has been implicated in oligodendrocyte pathologies 32, 33, 34. We further explored the AMPAR properties in hPSC‐derived oligodendroglial cells. Quantification of AMPAR subunit mRNA expression by quantitative real‐time PCR (qRT‐PCR) revealed that there was an increase in the GluA2, GluA3, and GluA4 mRNA expression levels relative to GluA1 in week 3 O4^+^‐oligodendrocytes compared with the levels observed in OPCs cultures in which OL differentiation was inhibited by absence of mitogen withdrawal (Fig. 6D). Our data indicate that GluA2 is the most prominently expressed AMPAR subunit in week 3 O4^+^‐oligodendrocytes.

GluA2 subunits are predominantly RNA edited in approximately 99% of central nervous system mRNA transcripts, where editing results in an arginine codon replacing a glutamine codon in the M2 re‐entrant loop region of the ion channel. The presence of the edited GluA2 [GluA2(R)] subunit in AMPAR complexes imparts low single‐channel conductance, low Ca^2+^ permeability, and insensitivity to polyamine‐mediated channel block 35. We examined the composition of AMPARs expressed by hPSC‐derived OPCs and oligodendrocytes by estimating the mean unitary single‐channel conductance using nonstationary fluctuation analysis (Fig. 6E, 6F 21). Unitary conductances were estimated by plotting current‐variance plots from AC‐ and DC‐coupled recordings of currents elicited by AMPA (10 μM) in the presence of cyclothiazide (10 μM) or in some recordings, AMPA (100 μM) alone (Fig. 6G). For the ES line, the mean AMPAR conductance of PDGFRα^+^‐OPCs decreased from 8.0 ± 0.2 pS (*n* = 14, *N* = 4) to 2.9 ± 0.1 pS in week 3 O4^+^‐oligodendrocytes (*n* = 4, *N* = 2, *p<*0.001, unpaired *t* tests, Fig. 6H). Equivalent data were also obtained from iPSC‐derived oligodendrocyte‐lineage cells (Fig. 6H). The data are consistent with the notion that GluR2(R) subunits are functionally upregulated in AMPARs expressed by oligodendrocytes. Supporting the idea that OPCs express a greater proportion of AMPARs that lack edited GluA2 subunits and oligodendrocytes AMPARs contain edited GluA2 subunits is the fact that the GluA2(R)‐lacking AMPAR antagonist polyamine, 1‐naphthyl acetyl spermine (NASPM, 36) caused substantial inhibition of AMPAR‐mediated currents in PDGFRα^+^‐OPCs (Fig. 6I), but not week 3 O4^+^‐oligodendrocytes (Fig. 6J, 6K).

### Oligodendrocytes Derived from Mutant *C9ORF72* Patients Express Intranuclear RNA Foci, but do not Exhibit Impairment in Maturation

**Figure 7:**
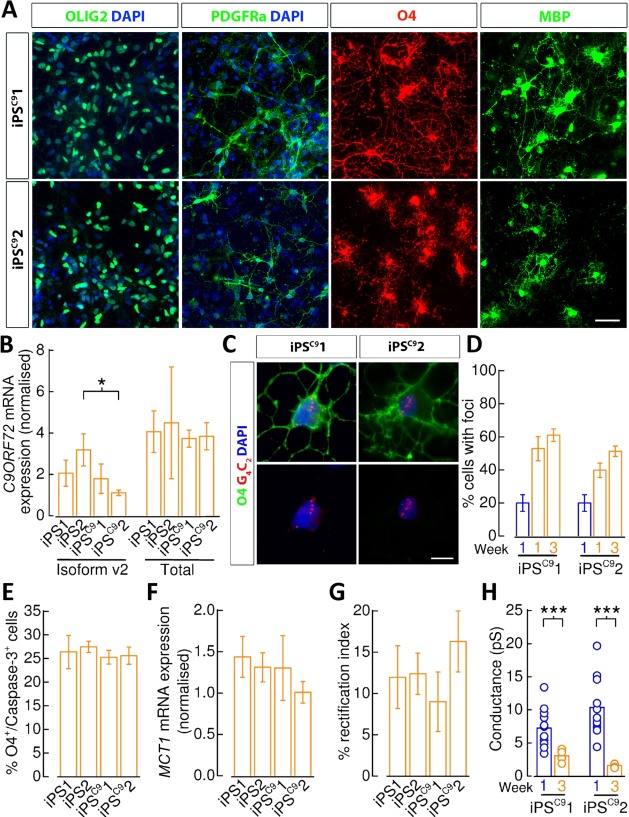
Oligodendrocytes derived from mutant *C9ORF72* patients. **(A)**: Representative staining of iPS^C9^1 and iPS^C9^2 cells 1 week post‐mitogen removal identifies OLIG2^+^‐progenitors, and cells expressing PDGFRα, O4, and MBP. Scale bar = 50 μm. **(B)**: MACS‐sorted week 3 *C9ORF72* mutant (iPS^C9^1 and iPS^C9^2) oligodendrocytes display comparable *C9ORF72*‐v2 and *C9ORF72*‐total (v1, v2, and v3 isoforms) expression compared with controls (iPS1 and iPS2) as showed by quantitative real‐time polymerase chain reaction (qRT‐PCR) (*N* = 5 for each line, unpaired *t* tests). **(C)**: Fluorescence in situ hybridization showing the presence of nuclear GGGGCC RNA‐containing foci in both iPS^C9^1 (*N* = 3) and iPS^C9^2 (*N* = 3) O4^+^‐oligodendrocytes. Scale bar = 10 μm. **(D)**: Percentage O4^+^‐oligodendrocytes derived from the iPS^C9^1 and iPS^C9^2 lines that express nuclear GGGGCC RNA‐containing foci at week 1 (iPS^C9^1/iPS^C9^2: *N* = 3/3) and 3 (iPS^C9^1/iPS^C9^2: *N* =  3/3). **(E)**: Flow cytometry quantification of O4^+^ and caspase3‐7^+^ cells showing no differences in cell death across the mutant (iPS^C9^1 and iPS^C9^2) and control (iPS1 and iPS2) lines in basal conditions (*N* = 3 for each line, unpaired *t* tests). **(F)**: MACS‐sorted week 3 *C9ORF72* mutant (iPS^C9^1 and iPS^C9^2) oligodendrocytes display no differences in MCT1 expression compared with controls (iPS1 and iPS2) as showed by qRT‐PCR (*N* = 5 for each line, unpaired *t* tests). **(G)**: Mean percentage reduction in rectification indices of currents recorded from week 3 O4^+^‐oligodendrocytes with respect to oligodendrocyte precursor cells (OPCs) (unpaired *t* tests). **(H)**: Mean AMPAR conductance for PDGFRα^+^‐OPCs (iPS^C9^1/iPS^C9^2: *n* = 12/11, *N* = 3/3) and O4^+^‐oligodendrocytes (iPS^C9^1/iPS^C9^2: *n* = 4/5, *N* = 2/1) derived from the iPS^C9^ lines. *, *p* < 0.05; ***, *p* < 0.001, respectively. Abbreviations: DAPI, 4',6‐diamidino‐2‐phenylindole; iPS, induced pluripotent stem cell; MBP, myelin basic protein; PDGFRα, platelet‐derived growth factor receptor alpha.

Having established benchmark physiological and maturation features of control OPCs and to assess the maturation profile of oligodendrocytes in a disease model we next derived oligodendroglia from hPSCs obtained from two ALS patients (iPS^C9^1 and iPS^C9^2) harboring mutations in the *C9ORF72* gene (Fig. 7A). Mutations in the *C9ORF72* gene are the most common manifestation of familial ALS and contribute to 10% of sporadic cases 37, 38.

The iPS^C9^ lines were previously determined to contain the 5′‐GGGGCC‐3′ hexanucleotide repeat expansions 39. We found the efficiency of oligodendrocyte‐lineage specification and differentiation from mutant lines comparable with control lines in respect to generation of enriched numbers of O4^+^‐labeled cells (*p* > 0.15), which exhibit high coexpression of MBP (*p* > 0.91) and little overlapping PDGFRα labeling (*p* > 0.82) versus control iPS lines (Supporting Information Fig. 1A, 1B). Similarly, few PDGFRα^+^‐OPCs persist in 3‐week old differentiation cultures versus control cells (*p* > 0.29; Supporting Information Fig. 1C).

Increased OPC proliferation has been observed in a mouse model of ALS expressing human mutant SOD1 and appears prevalent in ALS patient post mortem samples 3, 5. We, therefore, examined whether control and *C9ORF72* patient‐derived OPCs differed in their rates of proliferation using proliferative markers EdU and Ki67. Analysis of PDGFRα^+^/EdU^+^ and PDGFRα^+^/EdU^+^/Ki67^+^ however showed no consistent difference between control and patient‐derived lines (Supporting Information Fig. 1D).

We next investigated the consequence of *C9ORF72* mutation on differentiating oligodendrocytes. *C9ORF72* hexanucleotide repeat expansion results in reduction of C9ORF72 expression in the brain of patients carrying this mutation 37, 40, 41, 42. Therefore, we examined the expression of the C9ORF72 v2 isoform transcript, the most abundant of the isoforms, and total transcript levels (including isoforms v1, v2, and v3) in mutant oligodendrocytes. We observed no consistent reduction when compared with controls (Fig. 7B). The source of variability in these data may be that the detected C9ORF72 mRNA levels in oligodendrocytes were low when compared with iPSC‐derived neurons (relative fold expression with respect to neurons; v1, 11.0%; v2, 41.7%; v3, 3.9%), in agreement with the finding that *C9ORF72* is highly expressed in neurons compared with glia cells 43. Expanded repeats also generate repeat RNA that results in formation of RNA foci, characteristic of C9ORF72 pathology 37, 44, 45, 46. Using FISH, we observed that iPS^C9^‐derived O4^+^‐oligodendrocytes contain nuclear RNA foci (Fig. 7C, 7D). Foci were not observed in control O4^+^‐oligodendrocytes. In contrast we did not identify dipeptide repeat (DPR) proteins generated by non‐ATG translation of repeat RNA nor did we observe differences in TDP‐43 or p62 subcellular localization in *C9ORF72* O4^+^‐oligodendrocytes (data not shown). In view of recent findings suggesting morphological abnormalities in differentiating oligodendrocytes in mSOD1 experimental and pathological studies 3, we undertook Sholl analysis of week 1 oligodendrocytes, but this did not reveal any difference (data not shown). We address whether the mutation resulted in a cell viability phenotype by undertaking quantitative caspase3/7 counts in O4^+^‐cells using FACS and no difference was found between mutant and control (*p* > 0.38; Fig. 7E). Finally, noting that the oligodendrocyte‐specific lactate transporter monocarboxylate transporter‐1 (MCT1) has been described previously to be downregulated in post‐mortem samples of ALS patients and an ALS mouse model 3, 4, 5. Comparisons of MCT1 mRNA expression by qPCR in *C9ORF72* mutants with control week 3 oligodendrocytes also did not reveal a difference (*p* > 0.22, Fig. 7F).

Given that the presence of RNA foci in *C9ORF72* patient‐derived motorneurons is associated with changes in excitability 47 and maturation of mutant SOD1 oligodendrocytes is severely impaired in mouse 3, we next tested whether functional maturation of PSC‐derived oligodendrocyte‐lineage cells was affected by the presence of *C9ORF72* mutation. Whole‐cell capacitance and input resistance measurements did not reveal impairments in passive membrane properties versus controls (Supporting Information Fig. 1E, 1F). The percentage depression of rectification indices for week 3 O4^+^‐oligodendrocytes in both iPS^C9^ lines compared with those obtained from PDGFRα^+^‐OPCs showed no differences compared with control lines (iPS^C9^1, *p* > 0.30; iPS^C9^2, *p* > 0.27, Fig. 7G). Maturation to week 3 O4^+^‐oligodendrocytes was associated with an equivalent strong functional downregulation of *I*_*A*_ and *I*_*K*_ conductances but an increase in *K*_*ir*_‐channel expression (Supporting Information Fig. 1G–1I). *Na*_*V*_‐channel expression was present in all PDGFRα^+^‐OPCs (iPS^C9^1/iPS^C9^2, *n* = 5/5, *N* = 1/1), but not oligodendrocytes (iPS^C9^1/iPS^C9^2, *n* = 5/4, *N* = 1/1). In addition, an equivalent reduction in AMPAR unitary conductance (Fig. 7H) as they mature from OPCs to oligodendrocytes are seen in the iPS^C9^ lines compared with control lines. Collectively these findings are consistent with an absence of maturational deficit in mutant *C9ORF72*‐derived oligodendrocyte‐lineage cells.

## Discussion

Our data provide the first functional evidence of the maturation‐dependent changes in membrane conductances that occur in human oligodendrocytes as they differentiate from OPCs. We demonstrate that during differentiation and maturation human oligodendroglia recapitulate many of the changes in ion channel expression observed in rodent‐based systems. Using this platform, we show that the multiple properties of mutant *C9ORF72* ALS oligodendrocyte‐lineage cells, including membrane properties and maturation, are not impaired.

Our protocol efficiently generates cultures containing an enriched population of O4^+^‐ and MBP^+^‐oligodendrocytes within 1 week of hPSC‐OPC differentiation and is of an equivalent duration to that recently reported 17. The ability to propagate OPCs with FGF2 and PDGFα treatment and the reproducibility of the method across multiple hPSC lines confirms the suitability of the protocol for in vitro disease modeling studies. Critical to electrophysiological studies is the observation that >80% O4^+^‐oligodendrocytes co‐express MBP, but < 10% O4^+^‐cells coexpress PDGFRα. This allows live‐staining prior to electrophysiological analysis to identify and discriminate between OPCs and oligodendrocytes.hPSC‐derived OPCs display outwardly rectifying membrane currents and differentiation to oligodendrocytes results in a linearization of the membrane currents. These properties have been widely described in rodent oligodendroglial counterparts 23, 24, 25, in vitro mouse PSC‐derived OPCs 48 and integrated mouse PSC‐derived glial restricted progenitor cells that contain a proportion of OPCs 49, 50. These data indicate that comparable shifts in ion channel expression are occurring throughout human oligodendrogenesis in vitro. Our data show that the expression of voltage‐gated K^+^ channels, *I*_*A*_ and *I*_*K*_, is prominent in OPCs in all lines and that the reduction of expression in oligodendrocytes corresponds well to the shifts in membrane current properties. Such data are in accordance with previously observed developmental shifts in *I*_*A*_ and *I*_*K*_ channel expression in rodent oligodendroglial cells and also prominent expression of such membrane conductances in in vitro mouse PSC‐derived OPCs 48.

Spiking activity is observed in the majority of cells when OPCs are depolarized and is blocked by *Na*_*V*_‐channel blocker TTX. However, these spikes are not classified as bone fide action potentials due to their low amplitude. Oligodendrocytes did not exhibit any spiking activity, and we found that the functional expression of *Na*_*V*_ channels to be largely restricted to OPCs in agreement with reports from rodent oligodendroglial cells 24, 25, 51. In contrast to our data, hPSC‐derived OPCs have been previously shown to exhibit regenerative action potential firing in response to depolarization 52, however no native TTX‐sensitive current has been observed in in vitro murine PSC‐derived OPCs 48. It is important to highlight that numerous reports describe native OPCs being heterogeneous in their ability to display either spiking or nonspiking behaviors, which are likely to represent the differentiation/maturation status of the OPC rather than developmentally independent subclasses of OPC 26, 27, 28, 53. Since OPCs in this study are examined after a period of 1 week in oligodendrocyte differentiation medium, it may be argued that the early processes of oligodendrocyte differentiation may have already been initiated in OPCs leading to a reduced expression of *Na*_*V*_‐channels. Nonetheless the spiking activity and *Na*_*V*_‐channel current density data obtained from hPSC‐derived PDGFRα^+^‐OPCs are directly comparable with populations of NG2^+^‐cells in postnatal murine gray and white matter 26.

Our data indicate that *K*_*ir*_ channel expression is elevated in week 3 O4^+^‐oligodendrocytes above that seen in week 1 O4^+^‐oligodendrocytes and OPCs and correlates well with a hyperpolarization of the RMP of week 3 O4^+^‐oligodendrocytes. Indeed expression of inwardly rectifying *K*_*ir*_4.1 subunits in oligodendrocytes is critical to their maturation and their ability to myelinate in vivo 29. In addition, upregulation of *K*_*ir*_4.1 subunits has been proposed to allow OPCs in the adult brain to sense local changes in K^+^ concentrations generated by neuronal activity 30.

We next investigated the ability of PDGFRα^+^‐OPCs and O4^+^‐oligodendrocytes to express AMPARs, GABA_A_Rs, NMDARs, and GlyRs, each of which have previously been reported to be expressed in oligodendrocyte‐lineage cells 31. Our data indicate that the majority of cells respond to AMPA and GABA and the current density of AMPARsand GABA_A_Rs decreases with maturation of PDGFRα^+^‐OPCs to O4^+^‐oligodendrocytes. However, no responses to glycine or NMDA were observed in O4^+^‐oligodendrocytes. Importantly, the maintenance conditions for cultured oligodendrocyte‐lineage cells have been suggested to be a causal factor in reducing the functional expression of both NMDARs and GlyRs 31, 54.

Given the importance of AMPARs to oligodendrocyte‐lineage cell physiology and disease 32, 33, 34, we characterized the AMPAR composition. We initially determined that the expression of GluA2, 3 and 4 subunit mRNA was increased relative to GluA1 in oligodendrocytes compared with OPCs. The GluA2 subunit mRNA was the most prominently expressed subunit in the oligodendrocyte. In agreement with an increase in GluA2(R) expression from previous studies 55 (although see ref. 31), maturation of human oligodendrocytes is associated with a decrease in the unitary conductances of AMPARs. The most parsimonious explanation for this change in conductance is the increase in expression of edited GluA2(R) subunits expressed in AMPAR complexes as the conductance values for week 3 oligodendrocytes correspond well to those of recombinantly expressed heteromeric GluA2(R)‐containing AMPARs 56. In agreement with changes in unitary AMPAR conductance estimates, we observed a reduction in inhibition of AMPAR‐mediated currents by NASPM, an antagonist which blocks AMPARs that do not contain GluA2(R) subunits. While these data indicate AMPARs predominantly expressed in OPCs are GluA2(R)‐lacking and oligodendrocytes are GluA2(R)‐containing, our data suggest that small populations of AMPARs that possess GluA2(R)‐containing subunits in OPCs and a population of GluA2(R)‐lacking AMPARs in oligodendrocytes. Consistent with this notion is the finding that native rodent OPCs that have been shown to express both GluA2(R)‐lacking and GluA2(R)‐containing AMPARs, and in which their relative proportions are influenced in an activity‐dependent manner 57. Our data are consistent with numerous studies reporting an increase in functional expression of the GluA2(R) subunit in AMPARs expressed in rodent oligodendrocytes upon differentiation and maturation from OPCs 55, 58, 59. This developmental variability in AMPAR composition (and Ca^2+^‐permeability) is disease‐relevant and confers sensitivity of immature oligodendroglial cells to excitotoxic conditions 60. Adult human mature oligodendrocytes obtained from white matter post mortem samples however have been reported to express AMPARs at a low level and do not appear to express the GluA2 subunit mRNA 34. In this regard, AMPAR data in this study, therefore, are in direct contrast to human data, but are in good agreement with rodent AMPAR expression, composition and regulation in oligodendroglial cells.

Taken together these findings show species conservation of the defining physiological properties of oligodendrocyte‐lineage cells and crucially establish a platform to investigate disease related changes. Here, we examined the development and maturation of mutant *C9ORF72*‐patient derived OPCs given the accumulating evidence implicating oligodendrocyte dysfunction and pathology in sporadic and familial ALS including impaired maturation of oligodendrocytes in a mSOD1 mouse model 3, 4, 5, 61. Furthermore, the *C9ORF72* hexanucleotide expansion is implicated as a causal factor in abnormal neuronal excitability 47 and recent studies indicate that nuclear‐cytoplasmic transport is affected in motorneurons 62, 63. The precise function of C9ORF72 protein remains unknown although accumulating evidence implicates a gain of function mediated toxicity. This includes the absence of survival or motor deficits in a *C9orf72* knock‐out mouse model and a number of studies showing direct toxicity of RNA or translated dipeptide products 62, 63, 64.

We first confirmed no difference between control and mutant lines in respect to specification, OPC proliferation, maturation as measured by transition from PDGFRα^+^‐OPCs to O4^+^/MBP^+^‐oligodendrocytes or viability despite the widespread presence of RNA foci. Further pathological characterization revealed an absence of DPRs and TDP‐43 and/or p62 aggregates consistent with the recent pathological findings showing no clear association between the presence of RNA foci and TDP‐43 or p62 aggregation 65. Further interpretation of these findings is challenging as there is, to date, a limited literature describing oligodendrocyte specific pathological findings in ALS. In addition to reduced glial C9ORF72 expression compared with neurons there is well‐documented regional variation in pathology. For instance TDP‐43 and/or p62 inclusions are notably abundant in the cerebella and hippocampi 66, 67, 68. Together with the absence of cytotoxicity this suggests that the presence of the *C9ORF72* mutation does not confer detrimental effects on maturation or survival of oligodendrocytes.

In view of the multiple possible mechanisms of C9ORF72 mediated toxicity including non‐cell autonomous effects, it will be of interest to examine cocultures for motorneuron toxicity as has been shown for astrocytes 69. Our findings are specific to the process of oligodendrocyte‐lineage cell development and do not address myelination. In this respect, evaluation of mutant OPCs following transplantation into the non‐myelinating *Shiverer* mouse along with in vitro study of myelination upon coculture with neurons will be important. Finally, future studies may consider the extent to which genetic causes of ALS are heterogeneous with respect to the properties of oligodendrocytes.

## Conclusion

Our study demonstrates that the physiological maturation of oligodendrocytes from OPCs derived from human pluripotent stem cells is similar to that described for rodent models. Electrophysiological profiling of oligodendrocytes from OPCs derived from *C9ORF72* patients indicates that, despite the presence of RNA foci pathology, these cell populations display maturation changes in intrinsic membrane properties and AMPAR populations similar to those seen in control lines.

## Author Contributions

M.R.L. and D.M.: conception and design, collection and/or assembly of data, data analysis and interpretation, manuscript writing, final approval of manuscript; E.M.C., N.A.V., O.T.J., and B.T.S.: collection and/or assembly of data, data analysis and interpretation, final approval of manuscript; K.B.: collection and/or assembly of data, final approval of manuscript; D.S.: administrative support, collection and/or assembly of data, final approval of manuscript; C.E.S.: provision of study material or patients, manuscript writing, final approval of manuscript; P.C.K., G.E.H., D.J.A.W., and S.C.: conception and design, financial support, data analysis and interpretation, manuscript writing, final approval of manuscript. M.R.L. and D.M. contributed equally to this article.

## Potential Conflicts of Interest

The authors indicate no potential conflicts of interest.

## Supporting information

Additional Supporting Information may be found in the online version of this article

Supporting Information
