# Supplementary material for: Maturation and electrophysiological properties of human pluripotent stem cell‐derived oligodendrocytes
Source: Stem Cells. 2016 Jan 13;34(4):1040–53. doi: 10.1002/stem.2273 (PMC4840312; doi:10.1002/stem.2273)
Supplement: Supplementary file 1 — Supporting Information [file STEM-34-1040-s001.docx]

**Supplemental Text**

Maturation and electrophysiological properties of human pluripotent stem cell-derived oligodendrocytes

Matthew R. LIVESEY, Dario MAGNANI, Elaine M. CLEARY, Navneet A. VASISTHA, Owain T. JAMES, Bhuvaneish T. SELVARAJ, Karen BURR, David STORY, Christopher E. SHAW, Peter C. KIND, Giles E. HARDINGHAM, David J. A. WYLLIE and Siddharthan CHANDRAN.

**Material and Methods**

***Generation and validation of induced pluripotent stem cell lines*.** iPS1, iPS2 and iPS^C9^2 were reprogrammed from fibroblasts *via* ectopic expression of pluripotency transcription factors Oct4, Sox2, Klf4 and Myc *via* integrative retroviral methodology (Vectalys). iPS^C9^1 was reprogrammed *via* non integrative sendai methodology (Life technologies). Undifferentiated iPS cells were maintained on matrigel-coated (SLS, 354230; 1 in 60 dilution) plates and with Essential 8 media (A1517001, Invitrogen). Pluripotency was confirmed, as described previously [[1](#_ENREF_1), [2](#_ENREF_2)] by expression of pluripotency markers OCT4, SOX2, TRA-1–60 and NANOG and RT–PCR with three germ-layer differentiation confirmed by SOX1, Nestin, Brachyury, Eomes, FOXA2 and GATA-4 expression [[1](#_ENREF_1), [2](#_ENREF_2)]. All clones had a normal karytoype and confirmation of hexanucleotide 50-GGGGCC-30 repeat expansions in C9orf72 lines was shown by repeat prime PCR [[1](#_ENREF_1), [2](#_ENREF_2)].

***List of primary and secondary antibodies.*** Primary antibodies; OLIG2 (1:300, Millipore), Ki67 (1:100, Dako), MBP (1:50, rat, Abcam), GFAP (1:500, DAKO), GFAP-cy3 (1:500, mouse, IgG conjugated with Cy3; Sigma), K*_ir_*4.1 (1:200, Abcam). Secondary antibodies 1:1000; Alexa Fluor dyes, Alexa Fluor 555 Goat anti-rabbit IgG A21428, Alexa Fluor 488 goat anti-rabbit IgG (H+L) Cat. No. A11008, Alexa Fluor 647 goat anti-mouse IgM A21238, Alexa Fluor 555 Goat anti-Mouse IgG1 A21127, Alexa Fluor 555 goat anti-mouse IgG (H+L) A21422, Alexa Fluor 488 Donkey anti-Goat IgG A11055, Alexa Fluor 488 Goat anti-Rat IgG A11006.

***Flow Cytometry.*** Flow cytometry of oligodendrocytes was performed on a FACSCalibur (Becton Dickinson, San Jose, CA). Cells were lifted with Accutase (Sigma) and stained with primary antibody O4 1:500 (R&D Systems) and then secondary IgM (see above) 1:3,000. Secondary and unstained controls were also performed. Caspase3/7 (Life technology) was added at the last step of centrifugation and cell suspension was incubated at 37^o^C for 30 minutes. The cells were analyzed by forward and side scatter for Alexa Fluor 488 fluorescence through a 530 ± 30 nm band-pass and for Alex Fluor 647 through a 695 ± 40 nm band-pass. Unstained cells were used to set the background fluorescence; a false positive rate of 0.5% was accepted. FACS data was collected and analysed using Cellquest and Flowjo software.

***EdU labeling and detection.*** Following dissociation of oligospheres and plate down, mitotic cells were labeled with 10 μM ethynyl deoxy-uridine (EdU, Life Technologies) on day 3 for 24 hours. Media was replaced completely the next day and cells cultured for an additional 48 hours in the absence of EdU before fixation using 4% PFA (Sigma). For detection, cells were permeabilised using 0.1% saponin (Sigma) for 2 hours and detected using the EdU detection kit (Life Technologies) according to manufacturer’s instructions. Cells were also immunolabelled for PDGFRα as previously described to label for OPCs. Coverslips were washed and mounted using FluorSave (Merck).

***Quantitative RT-PCR****.* Protocols were performed as described previously [[3](#_ENREF_3)]. For qPCR studies, differentiated oligodendrocytes were lifted by using Accutase (Sigma) and MAC-sorted according to the manufacturer’s (Miltenyi Biotec) instructions using anti-O4 conjugated with magnetic beads. Forward and reverse sequences (5′→3′) of primers used were as follows:

*GRIA1*: TGCTTTGTCGCAACTCACAGA, GGCATAGACTCCTTTGGAGAAC;

*GRIA2*: CATTCAGATGAGACCCGACCT, GGTATGCAAACTTGTCCCATTGA;

*GRIA3*: ACCATCAGCATAGGTGGACTT, GGTTGGTGTTGTATAACTGCACG;

*GRIA4*: TTCCGAGCAGCGTGCAAATA, GCATTGGGGCTGGTGTTATGA;

*SLC16A1*: GACCTTGTTGGACCCCAGAG, AGCCGACCTAAAAGTGGTGG;

C9orf72 primers are from [[4](#_ENREF_4)].

***RNA fluorescence in situ hybridisation (FISH).*** FISH was performed using an Alexa 546-conjugated (GGCCCC)_4_ oligonucleotide probes (IDT). Briefly, cells on glass coverslips were fixed in 4% paraformaldehyde for 30 minutes, permeabilized in 70% ethanol at 4°C, incubated with 50% formamide/2X SSC for 10 min at room temperature, and hybridized for 2 h at 43°C with a Alexa 546-conjugated (GGCCCC)_4_ probe (0.16 ng/μl) in hybridization buffer consisting of 50% formamide, 2X SSC, 10% dextran sulfate, yeast tRNA (1 mg/ml), salmon sperm DNA (1 mg/ml) and 0.2% Tween-20. The cells were washed twice with 50% formamide/1X SSC for 30 minutes at 43°C and once with 2X SSC at room temperature for 30 minutes. Immunostaining was then performed as described after three washes in PBS.

**References**

1. Bilican B, Serio A, Barmada SJ et al. Mutant induced pluripotent stem cell lines recapitulate aspects of TDP-43 proteinopathies and reveal cell-specific vulnerability. **Proc Natl Acad Sci U S A***.* 2012;109:5803-5808.

2. Devlin AC, Burr K, Borooah S et al. Human iPSC-derived motoneurons harbouring TARDBP or C9ORF72 ALS mutations are dysfunctional despite maintaining viability. **Nat Commun***.* 2015;6:5999.

3. Livesey MR, Bilican B, Qiu J et al. Maturation of AMPAR composition and the GABA_A_R reversal potential in hPSC-derived cortical neurons. **J Neurosci***.* 2014;34:4070-4075.

4. Fratta P, Poulter M, Lashley T et al. Homozygosity for the C9orf72 GGGGCC repeat expansion in frontotemporal dementia. **Acta Neuropathol***.* 2013;126:401-409.

**Supplemental Figure**

**
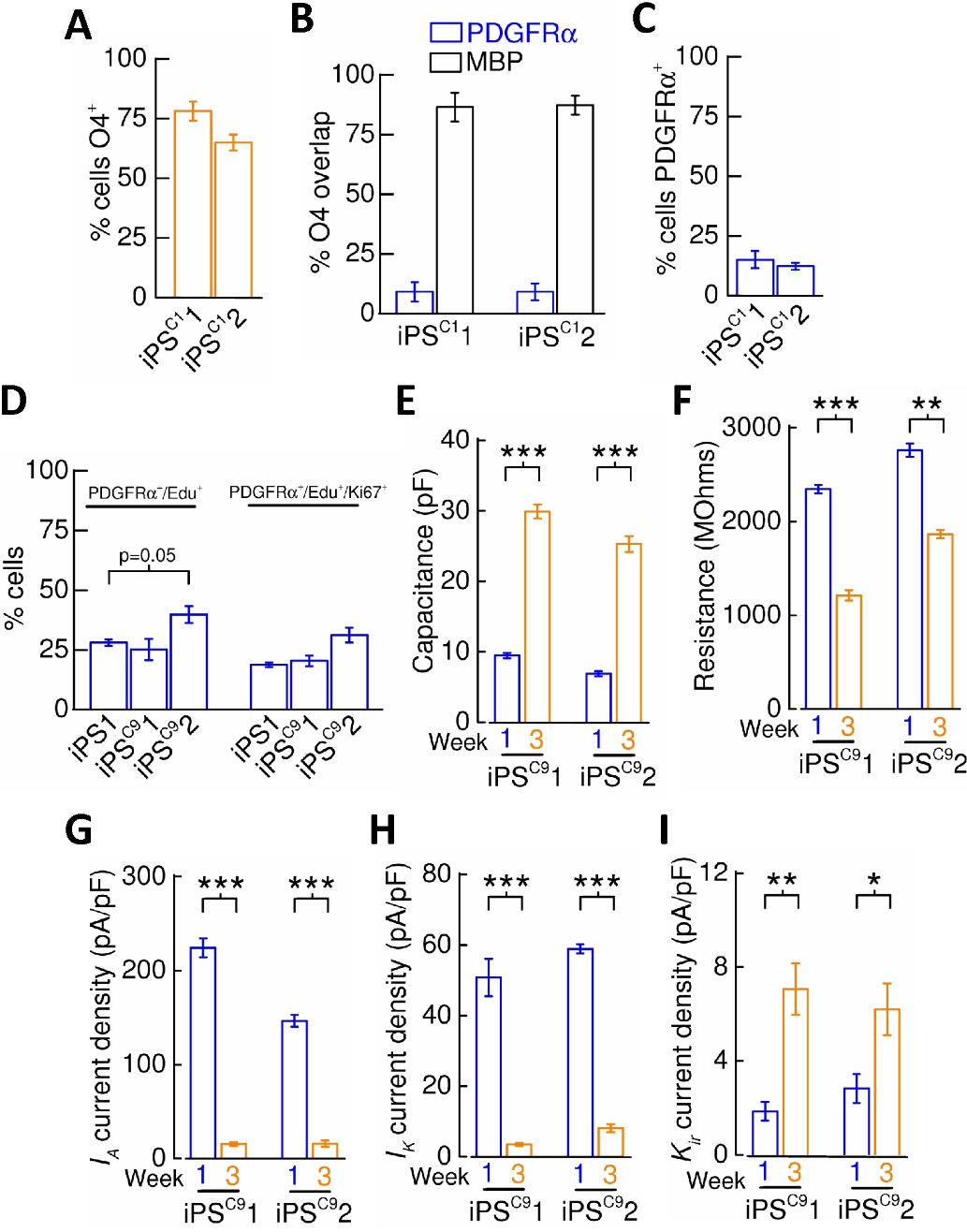
**

**Supplemental Figure.** The efficiency of oligodendrocyte lineage specification and differentiation from mutant C9orf72 lines (iPS^C9^1 and iPS^C9^2) was comparable to control lines. **A**, Percentage O4^+^-cells in Week 1 cultures (iPS^C9^1/iPS ^C9^2: *N* = 4/4). **B**, Percentage O4^+^-cells derived that express PDGFRα (iPS^C9^1/iPS ^C9^2: *N* = 5/5) and MBP (iPS^C9^1/iPS ^C9^2: *N* = 5/4). Equivalent cellular specification was observed across all lines. **C**, Percentage of PDGFRα^+^-cells (iPS^C9^1/iPS ^C9^2: *N* = 7/4). **D**, The proliferation kinetics of control and case iPS lines were examined. Cells were labeled using ethynyl deoxy-uridine (EdU) for 24 hours and 48 hours later cells were stained for PDGFRα, Ki67 and EdU. We found no consistent difference between lines in the percent of PDGFRα^+^/Edu^+^-cells (for iPS1 *versus* iPS^C9^2 *p*=0.05, *N*=3 each line) or in PDGFRα^+^/Edu^+^/Ki67^+^-cells (*p*>0.32, unpaired t-tests, *N*=3 each line). **E**, Mean whole-cell capacitance measurements for PDGFRα*^+^*-OPCs (iPS^C9^1/iPS^C9^2: *n*=9/6, *N*=3/1) and O4*^+^*-oligodendrocytes (iPS^C9^1/iPS^C9^2: *n*=17/18, *N*=3/2) derived from the iPS^C9^ lines. This data is not different *versus* controls (*p*>0.1, unpaired t-tests). **F**, Mean input resistance measurements for PDGFRα*^+^*-OPCs (iPS^C9^1/iPS^C9^2: *n*=14/11, *N*=3/2) and O4*^+^*-oligodendrocytes (iPS^C9^1/iPS^C9^2: *n*=17/18, *N*=3/2) derived from the iPS^C9^ lines. These measurements did not reveal any change *versus* controls (*p*>0.18, unpaired t-test). **G**, Mean *I_A_* current densities for PDGFRα*^+^*-OPCs (iPS^C9^1/iPS^C9^2: *n*=5/8, *N*=1/1) and O4*^+^*-oligodendrocytes (iPS^C9^1/iPS^C9^2: *n*=6/5, *N*=1/2) derived from the iPS^C9^ lines. **H**, Mean *I_K_* current densities for PDGFRα*^+^*-OPCs (iPS^C9^1/iPS^C9^2: *n*=8/7, *N*=1/1) and O4*^+^*-oligodendrocytes (iPS^C9^1/iPS^C9^2: *n*=5/5, *N*=1/1) derived from the iPS^C9^ lines. **i**, Mean *K_ir_* current densities for PDGFRα*^+^*-OPCs (iPS^C9^1/iPS^C9^2: *n*=5/5, *N*=1/1) and O4*^+^*-oligodendrocytes (iPS^C9^1/iPS^C9^2: *n*=5/4, *N*=1/1) derived from the iPS^C9^ lines. *, ** and *** denote significant differences with *p* values <0.05, 0.01 or 0.001, respectively.
